# Is full automation in ESR measurement clinically reliable? Revisiting a classic test

**DOI:** 10.1515/almed-2025-0191

**Published:** 2026-04-15

**Authors:** Alaitz Vélez de Mendizabal, Anna Marull Arnall, Leire Saiz Sierra, Maite Serrando Querol

**Affiliations:** Laboratori Territorial ICS-IAS Girona, Hospital Universitari Doctor Josep Trueta, Girona, Spain; Research Group of Clinical Anatomy, Embryology and Neuroscience (NEOMA), Department of Medical Sciences, University of Girona, Girona, Catalonia, Spain; Comisión de Hematología de la Sociedad Española de Medicina de Laboratorio (SEMEDLAB), Barcelona, Spain

**Keywords:** erythrocyte sedimentation rate (ESR), automation, harmonization

**Figure 1:**
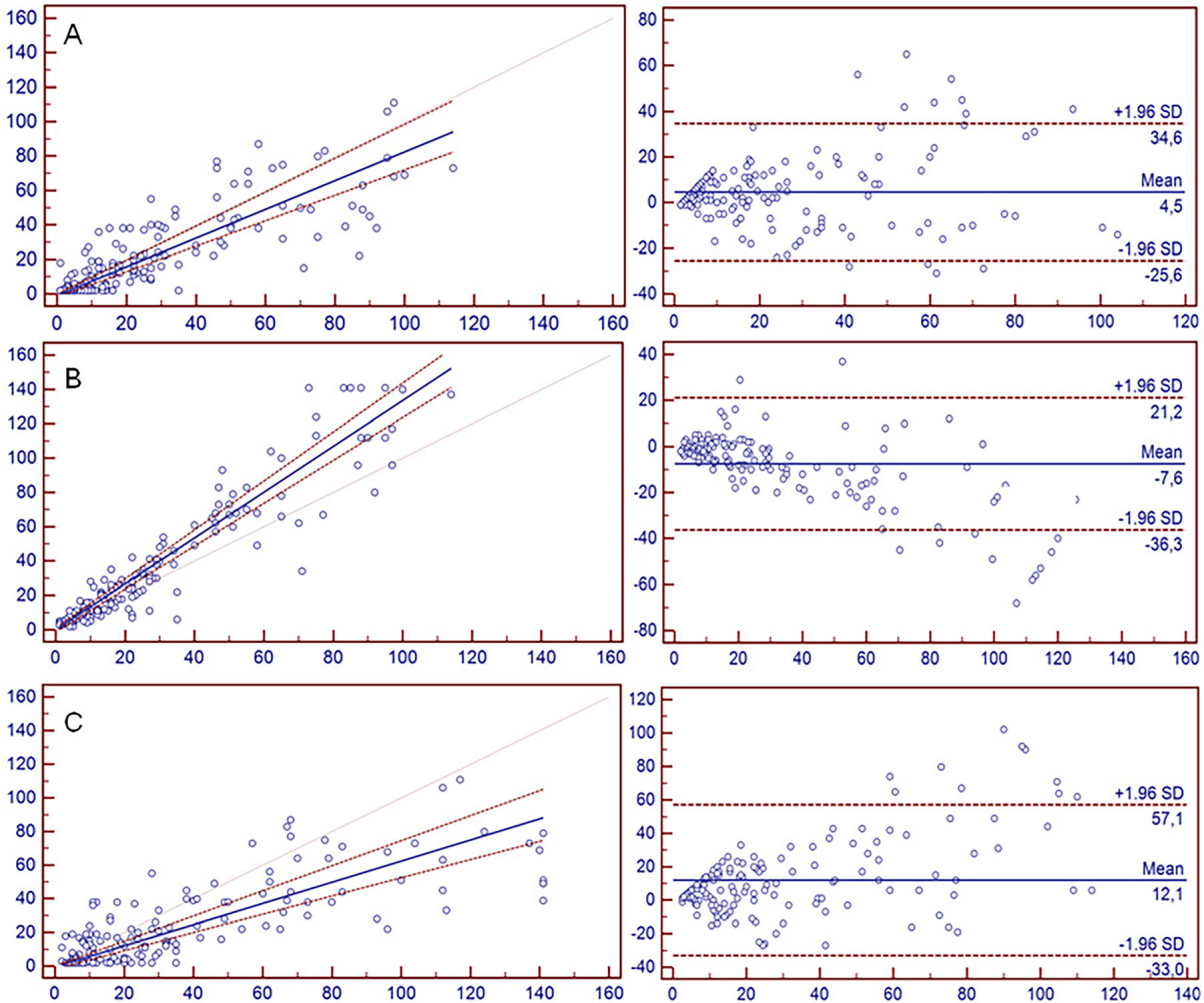
Comparison of erythrocyte sedimentation rate (ESR) measurement methods. (A) Left panel: regression between Westergren and Test1 2.0 (r=0.837, 95 % CI). Right panel: Bland-Altman plot showing a bias of −4.52 (95 % CI). (B) Left panel: regression between Westergren and Ves-matic Cube 80 (r=0.929, 95 % CI). Right panel: Bland-Altman plot showing a bias of +7.55 (95 % CI). (C) Left panel: regression between Test1 2.0 and Ves-matic Cube 80 (r=0.784, 95 % CI). Right panel: Bland-Altman plot showing a bias of −12.06 (95 % CI).

To the Editor,

The erythrocyte sedimentation rate (ESR) is a longstanding laboratory parameter used to measure the rate at which erythrocytes settle in anticoagulated blood over 1 h. Despite its well-known lack of specificity, ESR remains widely used in the evaluation and monitoring of inflammatory, infectious, neoplastic, and autoimmune diseases. Conditions such as temporal arteritis, polymyalgia rheumatica, rheumatoid arthritis, and Hodgkin lymphoma continue to rely on ESR as part of diagnostic and follow-up strategies. Its clinical value is enhanced when interpreted alongside C-reactive protein (CRP) and other acute-phase reactants, improving diagnostic accuracy and assessment of disease activity or therapeutic response [1], [2], [3] (Figure 1).

ESR is influenced by multiple physiological and hematological factors. Age and sex are consistent determinants, with higher values observed in older individuals and in females. Anemia accelerates sedimentation, whereas polycythemia slows it. Variations in erythrocyte morphology, hematocrit, and plasma protein composition – particularly increased fibrinogen and immunoglobulins – enhance rouleaux formation and sedimentation velocity. Pre-analytical and technical variables, including tube verticality, temperature, vibration, and dilution errors, may further affect results, underscoring the importance of standardization and cautious clinical interpretation [4], [5], [6].

The Westergren method, endorsed by the International Council for Standardization in Haematology (ICSH), remains the reference technique because of its reproducibility and diagnostic relevance. Using a 200 mm calibrated vertical tube, it evaluates erythrocyte aggregation, sedimentation, and packing phases, reflecting inflammatory changes through plasma protein–mediated rouleaux formation [2], 4], 5]. However, manual ESR testing is labor-intensive and operator dependent, leading to the development of automated analyzers to improve workflow efficiency and biosafety. Systems such as Test1 2.0 and Ves-matic Cube 80 employ optical or kinetic modeling of undiluted EDTA-anticoagulated blood and integrate efficiently into routine laboratory practice [1], 2], [6], [7], [8].

To assess analytical concordance with the Westergren method, ESR results from 166 patient samples (58 men, 108 women; age range 13–94 years) were analyzed using Test1 2.0, Ves-matic Cube 80, and manual Westergren. Mean ± SD ESR values were 22.28 ± 24.04 mm/h for Test1 2.0, 26.80 ± 26.98 mm/h for Westergren, and 34.36 ± 36.83 mm/h for Ves-matic Cube 80. Intraday precision of Test1 2.0 across nine samples (10–115 mm/h) showed coefficients of variation (CVs) between 0 % and 21 %. Interday precision using latex controls yielded CVs of 5.83 %, 4.23 %, and 2.88 %, supporting acceptable reproducibility for clinical use.

Passing–Bablok regression comparing Test1 2.0 with Westergren resulted in Y=0.834X − 1.006, with a 95 % confidence interval (CI) for the slope of 0.727–1.00, indicating a systematic error, particularly at higher ESR values. Spearman correlation was 0.837. Bland–Altman analysis demonstrated a mean bias of −4.52 mm/h, within clinically acceptable limits. Differences were expressed in absolute units (mm/h), consistent with standard ESR evaluation and clinical interpretation.

Ves-matic Cube 80 showed stronger correlation with Westergren (r=0.929); however, Passing–Bablok regression (Y=1.33X + 0.17; 95 % CI for slope 1.25–1.44) revealed a proportional bias, as the CI excluded the value 1. Bland–Altman analysis showed a mean positive bias of +7.55 mm/h, increasing with higher ESR values, consistent with proportional error.

Direct comparison between automated analyzers confirmed their lack of interchangeability. Test1 2.0 vs. Ves-matic Cube 80 yielded Y=0.63X – 0.55 (r=0.784), with a mean Bland–Altman bias of −12.06 mm/h, indicating both systematic and proportional errors. These discrepancies reflect fundamental differences in analytical principles: while Westergren measures the complete sedimentation process over 1 h, automated systems estimate ESR from early-phase sedimentation kinetics.

Recent advances have extended ESR assessment beyond the traditional one-hour endpoint. Sedimentation kinetics and machine learning approaches show promise in identifying acute infection and sepsis, although broader clinical validation and standardization are still required [9]. Despite the availability of more specific biomarkers such as CRP and procalcitonin, ESR retains value in chronic inflammatory and neoplastic conditions where longitudinal monitoring informs clinical decision-making [2], 3].

In conclusion, ESR remains a practical and clinically relevant marker. Automated systems improve efficiency and safety but require method-specific interpretation. Continued validation and harmonization efforts are essential to ensure reliable integration of ESR into modern laboratory practice. A limitation of this study is the exclusion of newer ESR estimation approaches integrated into modern hematology analyzers; therefore, the findings should be interpreted within the context of the evaluated systems.
